# CD44 as a Central Integrator of Inflammation and Fibrosis: From Molecular Signaling to Environmental Modulation

**DOI:** 10.3390/ijms26188870

**Published:** 2025-09-11

**Authors:** Agnieszka Pedrycz-Wieczorska, Patrycja Chylińska-Wrzos, Anna Grzywacz, Ewa Zieliński, Andrzej Bartosiński, Kornelia Kędziora-Kornatowska, Marta Lis-Sochocka, Paulina Mertowska, Sebastian Mertowski, Krzysztof Bojarski, Mansur Rahnama-Hezavah, Tomasz Urbanowicz, Ewelina Grywalska

**Affiliations:** 1 Faculty of Medicine and Health Sciences, University of Applied Sciences in Tarnow, 33-100 Tarnów, Poland; 2 Department of Histology, Embryology and Cytophysiology, Medical University of Lublin, 20-059 Lublin, Poland; Patrycja.Chylinska-Wrzos@umlub.edu.pl (P.C.-W.); Marta.Lis-Sochocka@umlub.edu.pl (M.L.-S.); 3 Independent Laboratory of Behavioral Genetics and Epigenetics, Pomeranian Medical University in Szczecin, 70-111 Szczecin, Poland; anna.grzywacz@awf.gda.pl; 4 Department of Medical Sciences and Public Health, Gdansk University of Physical Education and Sport, Kazimierza Gorskiego 1 St., 80-336 Gdansk, Poland; 5 Department of Emergency Medical Services, Collegium Medicum in Bydgoszcz, Nicolaus Copernicus University Toruń, 85-067 Bydgoszcz, Poland; 6 Faculty of Medicine, Collegium Medicum, Mazovian University in Płock, 09-402 Płock, Poland; a.bartosinski@mazowiecka.edu.pl; 7 Department of Geriatrics, Faculty of Health Sciences, L. Rydygier Collegium Medicum in Bydgoszcz, Nicolaus Copernicus University in Torun, Skłodowskiej Curie 9 Street, 85-094 Bydgoszcz, Poland; kornelia.kornatowska@cm.umk.pl; 8 Department of Experimental Immunology, Medical University of Lublin, 20-059 Lublin, Poland; sebastian.mertowski@umlub.pl (S.M.); ewelina.grywalska@umlub.pl (E.G.); 9 General Surgery Department, SP ZOZ in Łęczna, 21-010 Łęczna, Poland; 10 Department of Dental Surgery, Medical University of Lublin, 20-059 Lublin, Poland; 11 Cardiac Surgery and Transplantology Department, Poznan University of Medical Sciences, 61-701 Poznan, Poland

**Keywords:** CD44, fibrosis, hyaluronic acid (HA), myofibroblasts, environmental pollutants, epithelial–mesenchymal transition (EMT), tissue remodeling

## Abstract

CD44, a multi-isoform adhesion receptor for hyaluronic acid (HA), plays a crucial role in regulating cell interactions with the extracellular matrix, cell migration, differentiation, and survival in both physiological and pathological contexts. Accumulating experimental evidence suggests that CD44 is not merely a passive marker of mesenchymal cell activation but rather an active signaling hub driving fibrosis in many organs, including the lung, skin, heart, and liver. Its involvement in fibroblast differentiation into myofibroblasts, as well as induction of the invasive phenotype of these cells, shows striking analogies to the mechanisms of epithelial-to-mesenchymal transition (EMT) known from cancer progression. In this paper, we discuss both the molecular mechanisms of CD44-dependent signaling (including through EGFR, MAPK/ERK, CaMKII, lipid rafts, and Smad) and the influence of its modulation (knockout, antibodies, blockade of HA synthesis) on the course of fibrosis in in vitro and in vivo models. In addition, we present the influence of environmental pollutants—such as heavy metals, particulate matter, endocrine disruptors, and microplastics—on the activation of the HA-CD44 axis in connective tissue, with particular emphasis on their role in the induction of chronic inflammation, EMT, and extracellular matrix deposition. The collected evidence suggests that CD44 serves as a central integrator of inflammatory and fibrogenic signals, and its pharmacological modulation may represent a novel therapeutic strategy for treating fibrotic diseases and chronic inflammatory conditions.

## 1. Introduction

Connective tissue is a highly organized biological structure that plays a key role not only in providing support and mechanical integrity of organs, but also in maintaining the homeostasis of the cellular microenvironment. It participates in regenerative processes, the transmission of biochemical and immunological signals, and in creating a protective barrier against pathogenic factors. An essential element regulating these functions is surface receptors, which enable bidirectional communication between cells and the components of the extracellular matrix (ECM) [1,2,3]. A special place in this signaling network is occupied by CD44—a transmembrane glycoprotein belonging to the family of adhesion receptors, constituting the primary receptor for hyaluronic acid (HA) and also participating in interactions with other ECM components, such as osteopontin, collagens, fibronectin, and laminin [4,5,6]. The CD44 protein is characterized by a complex molecular structure and high heterogeneity resulting from alternative splicing, which leads to the formation of many isoforms (so-called CD44v). Individual variants differ in the length of the extracellular domain, the degree of glycosylation, and ligand affinity [7,8,9,10].

In physiological conditions, CD44 regulates several biological processes, including adhesion and migration of fibroblasts, lymphocytes, and progenitor cells, participates in the recruitment of immunocompetent cells, and also participates in mechanotransduction, ECM remodeling, and myofibroblast differentiation [11,12]. In pathological situations, such as chronic inflammation, organ fibrosis, or neoplastic progression, CD44 becomes a key mediator of disease progression, among others, through the activation of the phosphoinositide 3-kinase/protein kinase B (PI3K/AKT), mitogen-activated protein kinase (MAPK), and Ras homolog family member A/Rho-associated protein kinase (RhoA/ROCK) signaling pathways, as well as through interactions with transforming growth factor-beta (TGF-β) receptors, epidermal growth factor receptor (EGFR), and various growth factor binding molecules [13,14,15,16,17,18,19,20]. CD44, as a transmembrane glycoprotein acting as a cell surface receptor, plays a crucial role not only in binding hyaluronic acid but also in modulating signaling pathways, including the PI3K/Akt/mTOR pathway. Both PI3Kβ and mTOR play a crucial role in regulating key cellular processes, including survival, proliferation, metabolism, and the immune response. Under pathological conditions, particularly in inflammatory and neoplastic diseases, activation of these pathways via CD44 can lead to uncontrolled cell growth or dysregulation of the immune response. PI3Kβ is involved in, among other things, neutrophil activation and regulation of phagocytosis, while mTOR controls T-cell metabolism and inhibits autophagy, which is crucial for the course of infection and sepsis. Therefore, interactions of CD44 with extracellular matrix components may initiate signaling leading to pathological activation of PI3Kβ and mTOR, making these pathways potential therapeutic targets [21]. At the same time, recent years have seen a shift in attention to the anti-inflammatory potential of CD44, resulting from its interaction with molecules regulating the immune response, such as interleukin 37 (IL-37). In addition to its classic role as an HA receptor, CD44 can function as a co-receptor in a complex with IL-1R8—essential for IL-37 action. This anti-inflammatory cytokine inhibits the expression of numerous pro-inflammatory mediators (IL-1, TNF, IL-6, IL-17, CCL2), limits mTOR activation, and influences innate and adaptive immune cells, including macrophages, dendritic cells, and T lymphocytes. The dependence of IL-37 action on the presence of IL-1R8 and previous reports of this receptor’s interaction with CD44 indicate that CD44 may also participate in mechanisms of inflammatory response suppression. This suggests a dual function of CD44—as a promoter of pro-inflammatory signals and a potential stabilizer of the IL-37/IL-1R8 complex—which opens up new therapeutic possibilities in the context of diseases associated with immune system deregulation, such as sepsis, viral infections (e.g., COVID-19) or autoimmune diseases [22].

At the same time, increasing environmental pollution—including air, water, and soil—is associated with an increase in the incidence of immunological disorders and chronic tissue-based diseases. Particularly hazardous are particulate matter (PM2.5 and PM10), heavy metals (e.g., cadmium, lead, and arsenic), endocrine-disrupting compounds (EDCs, such as phthalates and bisphenol A), dioxins, and micro- and nanoplastics. Exposure to these substances can lead to oxidative stress, epigenetic modifications, damage to cell membranes, and activation of macrophages. As a result, ECM fragments are released, including degraded HA, which, as a DAMP (damage-associated molecular pattern) molecule, can strongly bind to CD44, activating pro-inflammatory, cytokine, and pro-fibrotic cascades [23,24].

These observations support the hypothesis that CD44 functions not only as an adhesion receptor but also as a sensor of environmental signals, integrating external stimuli with the cellular response and the remodeling of the tissue microenvironment [23]. CD44 expression and activity may undergo significant modifications under the influence of toxic environmental factors, which may lead to excessive activation of fibroblasts and myofibroblasts, disturbances in cell-ECM interactions, increased invasiveness of epithelial and neoplastic cells, and chronic inflammation in tissues exposed to toxins. The role of CD44 as an element of the “environment-microenvironment-cellular response” axis may vary depending on the type of exposure, isoform variant, tissue type, and cell population characteristics.

This paper aims to review the current literature on the expression, structure, and function of the CD44 protein, with a particular emphasis on its role as a key regulator of biological processes in connective tissue. An additional goal is to analyze the impact of environmental pollution factors, including chemicals, dust, heavy metals, and microplastics, on the regulation of the CD44 signaling pathway and its interactions with hyaluronan. Particular attention was paid to molecular mechanisms that may provide a basis for using CD44 as a biomarker of environmental exposure or a therapeutic target in diseases associated with chronic inflammation and connective tissue degradation.

## 2. Molecular Characteristics of the CD44 Protein: Structure, Isoforms, Biological Functions

The CD44 protein (also known as the CD44 antigen) is a cell surface glycoprotein encoded by the CD44 gene in humans (uniprot.org). It belongs to the family of CD (cluster of differentiation) particles, such as the CD44 antigen. The CD44 gene is located on chromosome 11 (region 11p13) and is sometimes referred to by various synonyms, including *LHR, MDU2, MDU3*, and *MIC4* [25]. The protein itself functions under many alternative names reflecting its diverse properties. The most common ones include: CD44 antigen (official name), CDw44, H-CAM (*Homing Cell Adhesion Molecule*), Hermes antigen, hyaluronate receptor, Epican, Extracellular matrix receptor III (ECMR-III) and terms indicating its proteoglycan character: heparan sulfate proteoglycan or chondroitin sulfate proteoglycan 8 (CSPG8) [26]. The CD44 protein is a significant component of the Indian blood group system (ISBT 023), which is one of the rare antigen systems of erythrocytes. The presence of specific antigens of this system—In^a^ and In^b^—is conditioned by the substitution of a single amino acid in position 46 of the CD44 polypeptide chain (arginine for In^b^, proline for In^a^). These antigens are encoded by allelic variants of the CD44 gene, located on chromosome 11 (11p13), and are primarily expressed on hematopoietic cells, including erythrocytes, lymphocytes, and monocytes. The Indian system exhibits autosomal codominant inheritance and may play a clinical role in transfusion medicine, particularly in cases of transfusion reactions or hemolytic disease of the newborn (Hemolytic Disease of the Newborn, HDFN). Although this system is rare in the general population, its presence has been reported to be more frequent in individuals of Indian and Pakistani descent, reflecting its historical name [25,27,28].

In the UniProt database, CD44 is listed under the accession number P16070 as a peer-reviewed entry within the UniProtKB/Swiss-Prot resource, designated with the entry name CD44_HUMAN. The canonical amino acid sequence of this protein includes 742 amino acid residues, while its molecular mass in the precursor form (including the signal sequence) is estimated at approximately 80–82 kDa [29]. CD44 is characterized by a high degree of structural heterogeneity, resulting from both the presence of numerous alternative isoforms (alternative splicing products) and various post-translational modifications, such as glycosylation, sulfation, and phosphorylation. This molecular variability is reflected in the diverse spectrum of biological functions that this protein performs in different cell types and physiological and pathological contexts. Many naturally occurring polymorphic variants have also been identified within the CD44 polypeptide sequence that can modify its three-dimensional structure, ligand binding ability, stability, and immunogenicity. Of particular importance are single amino acid substitutions (SAAS), some of which have been documented in both the UniProt database and the dbSNP set, and others have been associated with specific clinical phenotypes and disease states (Table 1) [12,30,31].

### 2.1. Biological Functions

CD44 is a transmembrane adhesion glycoprotein belonging to the class I receptor family, playing a crucial role in a wide range of biological processes, including both physiological mechanisms of homeostasis and pathological changes associated with inflammatory and neoplastic diseases [12,32]. The primary function of CD44 is to mediate interactions between cells and with the surrounding extracellular matrix (ECM). The primary ligand of this receptor is hyaluronic acid (HA), a key component of the ECM, responsible for maintaining the structural integrity and elasticity of tissues. In addition to HA, CD44 exhibits affinity for several other ECM components, including osteopontin, fibronectin, laminin, and collagens, as well as for proteolytic enzymes such as matrix metalloproteinases (MMP-2, MMP-9), which makes it an important integrator of signals from the extracellular environment [4,13,33,34,35,36,37,38,39,40,41,42] (Table 2).

In the context of the immune system, CD44 is involved in the adhesion and migration of leukocytes, enables the homing of T lymphocytes to lymphoid organs, and participates in the processes of their activation, survival, and proliferation [43,44,45]. The HCELL variant (*Hematopoietic Cell E-/L-selectin Ligand*) functions as a ligand for E- and L-selectins, enabling leukocytes to roll on the vascular endothelium, which is the first step in their recruitment to sites of inflammation [46,47,48,49]. CD44 also plays an essential role in hematopoiesis, progenitor cell migration, and bone marrow regeneration. Through its extracellular domain, it can bind cytokines, chemokines, proteases, and growth factors (e.g., Transforming Growth Factor Beta (TGF-β), Epidermal Growth Factor (EGF), and Vascular Endothelial Growth Factor (VEGF)), creating a local microenvironment that promotes the intensification of the cellular response [50,51,52,53]. The cytoplasmic domain of CD44, although short, binds to adaptor proteins (ezrin, radixin, and moesin), tyrosine kinases, and phospholipase C, initiating the activation of numerous signaling pathways, including the PI3K/AKT, MAPK/ERK, RhoA/ROCK, and GTPase pathways (Rac1 and Cdc42) [15,16,17,18,19,30,54,55,56,57,58,59,60,61].

**Figure 1:**
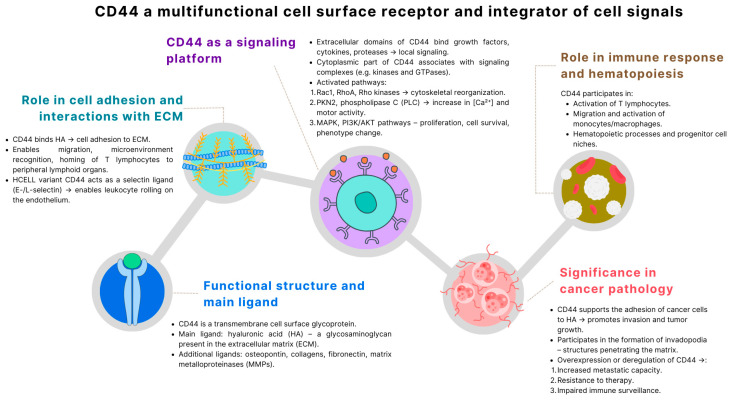
Multifunctional roles of CD44 in cell signaling, tissue homeostasis, immunity, and cancer progression (based on [62,64,65,66,70,71,73,75,76,77,78,79]). CD44, as a transmembrane glycoprotein, binds hyaluronic acid and additional ligands such as osteopontin, collagens, fibronectin, and MMPs, thereby integrating cell–ECM interactions. Beyond its structural role, CD44 acts as a signaling platform that organizes growth factors and adaptor proteins, activating pathways including PI3K/AKT, MAPK, and Rho-GTPases. These interactions support immune cell adhesion, migration, and hematopoiesis, while in cancer, CD44 facilitates invasion, metastasis, therapy resistance, and maintenance of tumor stem-like properties. Thus, CD44 emerges as a central regulator of adhesion, immune responses, tissue homeostasis, and malignant progression.

CD44 activation leads to the mobilization of calcium ions, reorganization of the actin cytoskeleton, and alterations in cell adhesion and morphology, which are essential components of processes such as migration, proliferation, differentiation, and inflammatory responses [62,63,64,65,66]. In pathological conditions, particularly in cancers, CD44 plays a crucial role in promoting the invasion, migration, and metastasis of cancer cells. As a marker of cancer stem cells (CSCs), CD44 facilitates the formation of invadopodia, specialized structures that enable ECM degradation and tissue penetration [67,68,69]. Altered CD44 expression, including the presence of specific alternative isoforms (e.g., CD44v6), correlates with increased tumor aggressiveness and a poorer clinical prognosis in numerous types of cancer, including colon, breast, and gastric cancers, as well as leukemias. Disorders in CD44 function or expression lead to a wide range of abnormalities, including impaired leukocyte migration, chronic inflammation, excessive activation of fibroblasts and myofibroblasts, pathological tissue fibrosis, and increased invasiveness of cancer cells. Due to the central position of CD44 as an integrator of environmental signals and a regulator of dynamic cellular interactions, this protein remains the subject of intensive research as a potential diagnostic and prognostic biomarker and therapeutic target in numerous disease entities (Figure 1) [4,70,71,72,73,74,75].

### 2.2. Subcellular Localization and Expression Profile of CD44 Isoforms

CD44 tends to preferentially localize in specialized domains of the cell membrane, particularly on the surface of microvilli and other membrane protrusions that increase the cell’s surface area of contact with its environment. In dynamic conditions, such as migration or cellular reorganization processes, this receptor concentrates in regions of active cell movement, including within the structures of membrane protrusions—lamellipodia and filopodia [80,81,82]. In vitro models, particularly in the context of wound healing, have demonstrated the colocalization of CD44 with actin filaments in the anterior regions of migrating cells, suggesting its involvement in regulating directional migration and cytoskeletal reorganization [55,83].

From the cytoplasmic side, the intracellular domain of CD44 interacts with the ERM family of anchoring proteins (ezrin, radexin, moesin), which connect it to the cortical layer of the actin cytoskeleton (Table 3). These interactions provide mechanical stability to the receptor complex while simultaneously enabling the transduction of signals from the cell surface to its interior, thereby activating, among others, the Rho- and Rac-dependent GTPase pathways. CD44 is also preferentially located in the so-called lipid rafts—microdomains of the cell membrane rich in cholesterol and sphingolipids, which promote the recruitment of signaling and adhesion proteins and participate in the modulation of signal transmission [52,54,56,84,85].

In terms of tissue expression, CD44 has a wide distribution and is constitutively expressed on the surface of many cell types, including both hematopoietic and non-hematopoietic cells [91,92,93]. The canonical form of CD44 (also known as CD44s, the standard form) is abundantly expressed in immune cells, including T lymphocytes, monocytic cells, and mesenchymal stromal cells. However, the *CD44* gene undergoes extensive alternative splicing, resulting in the emergence of numerous variant isoforms (CD44v), the expression of which is often tissue-specific and variable, depending on the physiological or pathological condition. For example, the isoform CD44v10, also known as the epithelial variant (CD44E), is highly expressed in epithelial cells and is described in detail in the oncology literature in the context of epithelial neoplasms (carcinoma). Its presence correlates with progression, invasiveness, and metastatic potential of many types of cancers, including breast, colon, and pancreatic cancers, making it a potential diagnostic and prognostic marker. A distinct CD44v expression profile is observed in neuroectodermal tumors, such as neuroblastoma, where reduced expression levels of specific variant isoforms have been identified, which may reflect tissue-dependent molecular differentiation and phenotypic plasticity of tumor cells [10,25,83,94,95].

### 2.3. Protein Structure and Isoforms

CD44 is a protein characterized by a differentiated domain architecture, the structure of which determines the receptor’s ability to participate in dynamic adhesion processes, signaling, and spatial organization of the cell (Table 4). The structure of the mature polypeptide, resulting from the cleavage of the signal sequence directing translocation to the endoplasmic reticulum, includes 722 amino acids and consists of three main functional segments: the extracellular domain, the transmembrane segment, and the cytoplasmic domain [96,97].

The most extensive part of the molecule is the extracellular domain (residues 21–649), which is responsible for direct interaction with extracellular matrix ligands. Its N-terminal part contains a conserved LINK-type domain, with a structure homologous to glycosaminoglycan-binding proteins, which ensures high affinity for HA and ligand selectivity. In the further part of the extracellular domain, there are regions encoded by alternative exons, the presence of which is the result of the differential splicing mechanism. These fragments, often rich in hydroxyl residues (serine, threonine) and proline residues, are potential sites of post-translational modifications, including O-glycosylation and the attachment of glycosaminoglycan chains (e.g., chondroitin sulfate), which functionally classifies CD44 as a transmembrane proteoglycan. Additionally, the presence of conserved N-glycosylation sites on asparagine residues affects the protein’s proper folding, conformational stability, and correct localization in the plasma membrane [25,98,99].

The extracellular domain is separated from the cytoplasmic domain by a transmembrane segment (residues 650–670), which takes the form of a hydrophobic α-helix stably anchored in the lipid bilayer. This region is rich in apolar residues, such as leucine, alanine, and valine, which are responsible for the correct placement of the molecule in the membrane and the orientation of the domains. Specifically, the N-terminus is located on the external side, while the C-terminus is positioned in the cytoplasm. The C-terminal cytoplasmic domain (residues 671–742), devoid of a permanent tertiary structure (intrinsically disordered region), shows high functional flexibility and plays a central role in signal transduction. It contains basic motifs (rich in lysine and arginine) and serine-threonine phosphorylation sites, regulating signaling activity and the stability of interactions with molecular partners. Of key importance are the bindings to adaptor proteins of the ERM family (ezrin, radexin, moesin), which enable anchoring of the receptor in membrane domains with high signaling activity (e.g., lipid rafts) and physical coupling with the actin cytoskeleton. Through these interactions, CD44 integrates signals from the cellular environment, influencing the reorganization of cytoskeletal structures, cell polarity, and its motility and invasiveness [25,30,100,101,102].

#### 2.3.1. Alternative Splicing of the *CD44* Gene and Characterization of Selected Isoforms

**Figure 2:**
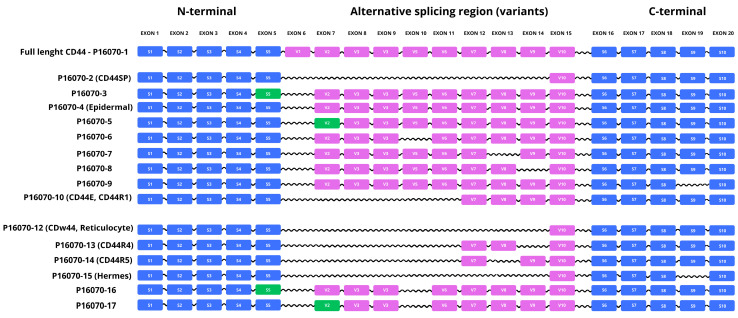
Structural organization of CD44 isoforms resulting from alternative splicing. Diagram shows the canonical CD44 isoform (P16070-1) and numerous alternatively spliced variants, showing their exonic composition within the N-terminal domain, the variable region (alternative splicing), and the C-terminal domain. Exons encoding the constant N-terminal region (S1–S5, blue) are present in all major variants, while the central variable region (V2–V10, purple/green) reflects the high splicing diversity. The C-terminal region (S6–S10, blue) is conserved in most isoforms, accounting for the transmembrane and cytoplasmic domains. Green labels indicate exons with alternative splice donor/acceptor sites. Individual isoforms (e.g., CD44v2, CD44v4, CD44v6, CD44R1, Hermes) differ in the characteristic presence or absence of selected exons, which determines their diverse biological functions, such as adhesion, cell migration, regulation of the immune response or cancer progression (based on [106,108,109,110,111,112,113]).

The *CD44* gene is subject to complex transcriptomic regulation, including intensive alternative splicing, which is a key mechanism generating its structural and functional diversity. This process involves the variable inclusion of up to 10 of the 19 exons encoding the extracellular domain, as well as the use of internal splicing sites and alternative splicing of the C-terminal region, including the cytoplasmic tail. As a result of such multivariability, numerous isoforms are formed—both membrane, with different lengths and compositions of the extracellular domain, and soluble forms, lacking the transmembrane segment, which can be secreted into the extracellular space. At least 19 different isoform variants of the CD44 protein have been described in the UniProt database, although some of them remain unconfirmed at the protein level in proteomic studies (Table 5, Figure 2) [99,100,101,103].

The reference isoform is considered to be P16070-1, which includes the complete set of possible exons and encodes a 742-residue polypeptide of approximately 82 kDa. Although sometimes referred to as CD44H or the “full” form of CD44, it is not the same as the so-called standard isoform (CD44s), which in cell biology nomenclature means a simplified, shortened version devoid of variant exons. CD44s corresponds to the P16070-2 isoform and is widely expressed in hematopoietic cells, such as T lymphocytes, where it functions as a basal adhesion receptor. In addition to these two basic variants, numerous isoforms with specific tissue expression or characteristic of pathological conditions have been identified. An example is the P16070-10 isoform, known as CD44E, which occurs in epithelial cells and lacks exons 6–11; this variant often correlates with the presence and aggressiveness of epithelial neoplasms. Another isoform, CD44R2 (P16070-11), lacks exons 6–13, whereas CDw44 (isoform 12) includes a form that excludes exons 6–14, which was initially detected in reticulocytes. Another interesting variant is CD44R5 (isoform 14), in which sequences encoded by exons 6–11, 13, and 14 are excluded, which significantly affects the topology of the extracellular domain. In turn, the Hermes isoform (P16070-15), which lacks exons 6–14 and 19, has been described as an antigen involved in lymphocyte homing—the ability of lymphocytes to migrate and colonize lymphoid organs selectively. Of particular note is the P16070-19 isoform, known as CD44RC, which is a soluble form lacking a transmembrane segment that can be released into the extracellular environment; it has an increased ability to bind hyaluronic acid and can act as a so-called decoy receptor, modulating the availability of the ligand for other forms of CD44. The expression of individual CD44 isoforms is precisely regulated in a manner dependent on the stage of development, cell type, and physiological or pathological condition of the organism. In healthy somatic tissues, the standard form of the protein dominates, while isoforms containing additional exons, collectively referred to as CD44v (variant), appear with greater frequency in pathological conditions, especially in the context of cancer. Molecular observations indicate that variants containing exons v6 and v10 correlate with the intensity of invasive and metastatic features of cancer cells, which suggests their significant participation in the clinical progression of the disease [101,102,103,104,105,106,107].

**Table 5:** Summary of CD44 Isoforms (based on [106,108,109,110,111,112,113]). The table provides an overview of canonical and alternative CD44 isoforms generated by extensive alternative splicing events. Each isoform differs in amino acid length, molecular mass, isoelectric point, and hydropathic profile, reflecting distinct structural and functional characteristics. Specific deletions or amino acid substitutions associated with exon skipping or alternative splice donor/acceptor usage determine ligand-binding capacity, subcellular localization, and signaling potential. Collectively, the diversity of CD44 isoforms underlies their specialized roles in processes such as immune cell homing, epithelial differentiation, tissue remodeling, and cancer progression.

Isoform	Length (aa)	Mass (Da)	pI	% Hydrophilic	% Hydrophobic	Description
P16070-1	742	81,538	4.98	57.95%	42.05%	Canonical isoform.
P16070-2 (CD44SP)	29	3327	9.37	33.33%	66.67%	Lacks exons 6–14. Differences: 23–29: DLNITCR → GVGRRKS; 30–742: deleted.
P16070-3	711	77,983	5.5	58.23%	41.77%	Alternative splice donor/acceptor in exon 5. Differences: 192 G→A; 193–223 deleted.
P16070-4 (Epidermal)	699	76,612	4.94	68.24%	31.76%	Lacks exon 6. Differences: 223 T→S; 224–266 deleted.
P16070-5	734	80,790	4.98	67.71%	32.29%	Alternative splice donor/acceptor in exon 7. Differences: 266–273 deleted.
P16070-6	699	76,705	4.95	67.38%	32.62%	Lacks exon 10. Differences: 385 I→T; 386–428 deleted.
P16070-7	713	78,446	5.05	60.17%	39.83%	Lacks exon 13. Differences: 506 Q→R; 507–535 deleted.
P16070-8	674	74,388	4.88	60.83%	39.17%	Lacks exon 14. Differences: 536 N→R; 537–604 deleted.
P16070-9	675	74,196	4.91	61.19%	38.81%	Lacks exon 19. Differences: 675 R→S; 676–742 deleted.
P16070-10 (CD44E, CD44R1, Epithelial, Keratinocyte)	493	53,411	5.02	66.13%	33.67%	Lacks exons 6–11. Differences: 223 T→N; 224–472 deleted.
P16070-11 (CD44R2)	429	46,565	5.24	56.88%	43.12%	Lacks 223–535 region.
P16070-12 (CDw44, Reticulocyte)	361	39,416	5.04	50.97%	49.03%	Lacks exons 6–14. Differences: 223 T→R; 224–604 deleted.
P16070-13 (CD44R4)	425	46,261	4.86	59.06%	40.94%	Lacks exons 6–11 and 14. Differences: 223 T→N; 224–472 and 537–604 deleted; 536 N→R.
P16070-14 (CD44R5)	396	43,169	4.99	59.60%	40.40%	Lacks exons 6–11, 13 and 14. Differences: 223 T→N; 224–472, 507–535 and 537–604 deleted; 506 Q→R; 536 N→R.
P16070-15 (Hermes)	294	32,075	4.86	54.76%	45.24%	Lacks exons 6–14 and 19. Differences: 223 T→R; 224–604 and 676–742 deleted; 675 R→S.
P16070-16	668	73,150	5.03	60.63%	39.37%	Alternative splice donor/acceptor on exon 5; lacks exon 10. Differences: 192 G→A; 193–223 and 386–428 deleted; 385 I→T.
P16070-17	691	75,957	4.95	67.15%	32.85%	Alternative splice donor/acceptor on exon 7; lacks exon 10. Differences: 266–273 and 386–428 deleted; 385 I→T.
P16070-18	340	37,278	5.15	61.47%	38.53%	Differences: 223 T→R; 224–604 and 605–625 deleted.
P16070-19 (CD44RC)	139	15,635	7.73	61.87%	38.13%	Soluble isoform; enhanced HA binding. Differences: 78–139 replaced with SLHCSQQSKK...QGVVRNSRPVYDS; 140–742 deleted.

#### 2.3.2. Protein Interactions and Complexes

**Figure 3:**
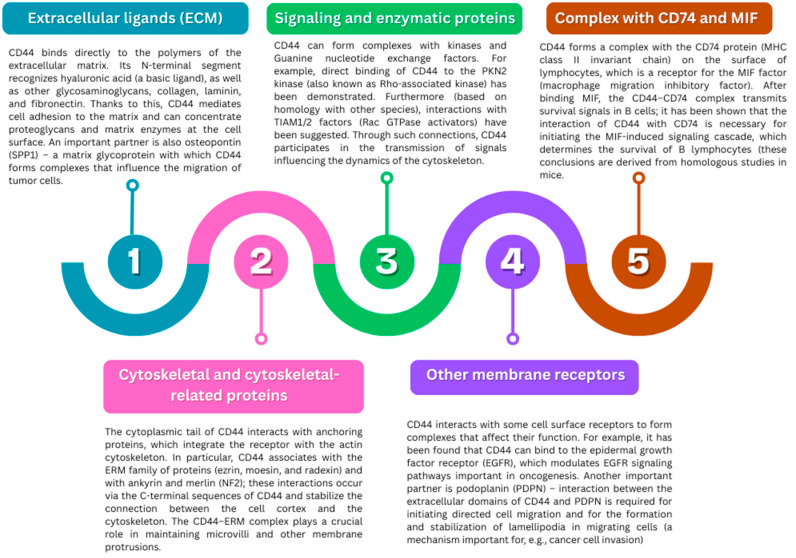
Functional interactions of CD44 with components of the extracellular matrix, intracellular signaling molecules, and membrane-associated partners (based on [12,25,120,121,122,123,124,125,126,127,128,129]). The figure summarizes the diverse molecular interactions of CD44, highlighting its role as a multifunctional hub. In the extracellular space, CD44 binds to hyaluronic acid, collagens, laminin, fibronectin, and osteopontin, mediating adhesion, migration, and tumor cell invasion. Through its cytoplasmic domain, CD44 associates with ERM proteins (ezrin, moesin, radixin) and NF2/merlin, linking the receptor to the actin cytoskeleton and supporting the dynamic regulation of microvilli and cell polarity. Additionally, CD44 interacts with signaling and enzymatic proteins such as kinases (e.g., PKN2, Rho-GTPases), transmitting signals that regulate cytoskeletal organization and cell motility. Cooperation with other membrane receptors, including EGFR and podoplanin (PDPN), further integrates CD44 into signaling networks that control proliferation, migration, and cancer invasion. Finally, the complex of CD44 with CD74 and MIF illustrates its role in immune cell survival and regulation, underscoring the receptor’s significance in inflammation, immunity, and tumor progression.

CD44 acts as a dynamic transmembrane receptor that integrates signals from the extracellular and intracellular spaces. Its extracellular domain enables the binding of extracellular matrix components, such as hyaluronic acid, collagens, osteopontin, or fibronectin, which promote cell adhesion, migration, and communication with the environment [107,114]. The cytoplasmic domain of CD44 interacts with cytoskeleton-anchoring proteins (e.g., ERM, merlin), kinases, and GTPase regulatory proteins, enabling the activation of intracellular signaling pathways. CD44 also forms complexes with other membrane receptors (e.g., EGFR, PDPN) and coreceptors (CD74), which affect cell proliferation, survival, and motility in response to environmental signals [108,109,115,116,117,118,119]. Through these complex interactions, CD44 acts as a central signaling platform, integrating the mechanisms of adhesion, migration, cytoskeletal regulation, and cellular response to external stimuli (Figure 3).

#### 2.3.3. Post-Translational Modifications (PTMs)

The CD44 protein is subject to numerous post-translational modifications (PTMs) that are crucial for its localization, stability, molecular interactions, and biological functions. The most important of these include glycosylation, phosphorylation, and proteolysis, as well as rarer modifications such as ubiquitination. Variability in the type, location, and intensity of these modifications contributes to the considerable functional heterogeneity of CD44, especially in the context of tumor pathologies and interactions with the extracellular matrix (Table 6).

## 3. The Role of CD44 in Pathologies

In addition to its physiological functions, CD44 plays a crucial role in the pathogenesis of neoplastic, autoimmune, and inflammatory diseases, where its expression and function change. Different CD44 isoforms (standard and variant) play various roles depending on the disease context [12,129,141,142,143,144] (Table 7).

In the context of neoplastic diseases, CD44 acts in a multidirectional manner, promoting the malignant phenotype of cells. Overexpression of this protein is common in tumors showing features of invasiveness, resistance to treatment, and high metastatic potential. CD44-positive cells are characterized by the activation of signaling pathways that increase their survival, migration, and resistance to environmental stress, especially in hypoxic conditions and during cytotoxic therapies. CD44 interacts with the tumor microenvironment, influencing its remodeling, and also supports the formation of pre-metastatic niches through adhesion to the extracellular matrix and localization of proteases, such as MMP-9, on the cell surface [145,146].

Variant isoforms, including CD44v6 and CD44v3, play an additional role—they participate in angiogenesis, activation of MET and VEGFR2 receptors, and binding of growth factors using heparan sulfate residues [147,148]. Switching splicing from the standard form (CD44s) to the variant or vice versa is closely correlated with the stage of cancer progression, e.g., in colon or liver cancer. Overexpression of CD44, regardless of its form, is often associated with shorter patient survival, more advanced TNM stages, and a higher risk of metastases, making it not only a prognostic marker but also a potential target for molecularly targeted therapy [149,150,151]. In pathologies with an immune basis, CD44 plays a crucial role in the pathomechanisms of chronic inflammation, primarily by facilitating the migration, activation, and retention of leukocytes in affected tissues. In rheumatoid arthritis, increased expression of CD44—both on immune cells and synovial fibroblasts—promotes the perpetuation of inflammatory infiltrate and cartilage degradation. Antibodies against CD44 reduce the migration capacity of fibroblasts and alleviate symptoms of inflammation in animal models [149,150,151,152,153,154,155,156].

In lupus nephritis (LN), the presence of CD44 on tubular epithelial cells and infiltrating leukocytes is associated with increased inflammation and fibrosis. Inhibition of CD44 activity in a mouse model led to reduced proteinuria, collagen deposition, and lymphocytic infiltration [140,157,158]. Furthermore, serum levels of the soluble form of CD44 (sCD44) are a useful biomarker of disease activity, with its increase preceding clinical exacerbations, suggesting its potential use in monitoring patients. In inflammatory bowel disease (IBD), such as Crohn’s disease and ulcerative colitis, increased expression of CD44 variants—particularly CD44v3, CD44v6, and CD44v7—has been reported in the intestinal mucosa. Functionally, CD44v7 interactions with osteopontin promote IL-6 production by macrophages, which in turn supports inhibition of T regulatory cell differentiation and aggravates inflammation. Blocking this interaction in animal models reduces the severity of colitis and restores immune balance. As a result, the CD44v7–osteopontin pathway represents a promising target for novel targeted therapies in IBD [129,141,142,143,144,145,159].

**Table 7:** Pathological role of CD44 in selected disease entities (based on [74,135,146,147,160,161,162,163,164,165,166,167,168,169,170,171,172,173,174]). The table highlights the diverse pathological functions of CD44 across malignant and inflammatory diseases. Distinct isoforms, including CD44s, CD44v, and soluble CD44, contribute to tumor initiation, invasion, therapy resistance, and immune dysregulation through interactions with HA, osteopontin, growth factor receptors, and proteases. These mechanisms involve activation of STAT3, PI3K/Akt, and other signaling cascades, supporting processes such as epithelial–mesenchymal transition, fibrosis, or chronic inflammation. Clinically, CD44 expression and isoform profiles serve as prognostic and predictive biomarkers and represent potential therapeutic targets in both oncology and autoimmune disorders.

Disease/Cancer Type	Role of CD44	CD44 Isoforms	Mechanism of Action	Clinical Significance
Breast cancer	CSC marker, EMT, therapy resistance	CD44s, CD44^high	STAT3 and PI3K/Akt activation; splicing toward CD44s	Poor prognosis, tumor recurrence
Pancreatic cancer	Invasion, metastasis	CD44v6	MMP-9 localization, interactions with HA	Promotes metastasis, reduces overall survival (OS)
Lung cancer	Proliferation, chemoresistance	CD44v	MET/VEGFR2, PI3K/Akt signaling	Accelerated tumor growth
Rheumatoid arthritis (RA)	Leukocyte adhesion, fibroblast activation	CD44v (various)	HA interactions, pannus formation	CD44 blockade reduces inflammation
Lupus nephritis (LN)	Inflammation, renal fibrosis	CD44s, sCD44	CD4+, CD19+ recruitment, fibroblast activation	Disease activity biomarker, therapeutic target
Crohn’s disease	IL-6 production, Treg deficiency	CD44v7	Interaction with osteopontin	CD44v7 blockade protects against colitis

### 3.1. CD44 as a Regulator of Tissue Fibrosis and Myofibroblast Differentiation

**Figure 4:**
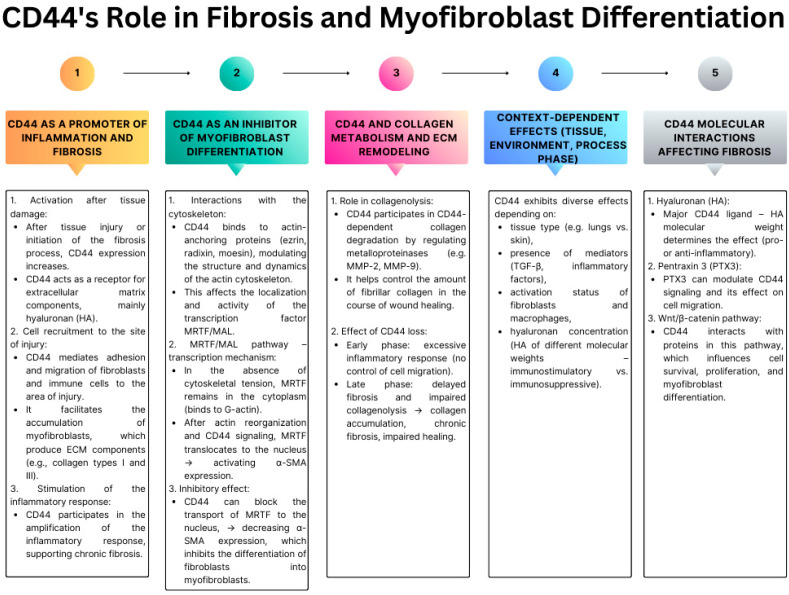
The multifunctional role of CD44 in fibrosis and myofibroblast differentiation (based on [175,179,180,181,182,183,184,185,186,190]). The figure illustrates the pleiotropic functions of CD44 during fibrotic processes, emphasizing its dual role as both a promoter of inflammation and an inhibitor of myofibroblast differentiation. CD44 regulates fibroblast and immune cell recruitment, ECM remodeling, and collagen metabolism through interactions with hyaluronic acid, ERM proteins, and metalloproteinases. Its activity is highly context-dependent, influenced by tissue type, molecular environment, and the presence of mediators such as TGF-β or inflammatory factors. Moreover, specific molecular interactions—including hyaluronan binding, pentraxin 3–dependent signaling, and Wnt/β-catenin pathway regulation—shape CD44’s impact on cell migration, survival, proliferation, and myofibroblast differentiation, ultimately determining the balance between tissue repair and pathological fibrosis.

Fibroblast differentiation into myofibroblasts is an essential step in the repair and fibrotic processes that occur in response to tissue damage. Myofibroblasts, through the expression of α-smooth muscle actin (α-SMA) and contractile properties, are capable of remodeling the ECM and restoring tissue integrity (Figure 4) [175,176,177,178]. However, their sustained activation and excessive numbers underlie many fibrotic pathologies, such as idiopathic pulmonary fibrosis, liver cirrhosis, and restrictive cardiomyopathies. The central factor inducing this phenotype is TGF-β1, which activates the canonical Smad2/3 signaling pathway, leading to the expression of myofibroblast-specific genes. However, increasing evidence suggests that the presence of TGF-β1 alone is insufficient to activate the differentiation program fully. Simultaneous signaling from HA and its primary receptor, the glycoprotein CD44, which acts as a coreceptor to amplify and modulate signal transmission, is necessary [179,180,181]. CD44, as an HA receptor, participates in the formation of a dynamic signaling structure on the surface of fibroblasts. The presence of high-molecular-weight HA and its binding to CD44 enables the localization of the receptor complex in lipid rafts of the cell membrane—specialized domains rich in cholesterol and sphingolipids that act as signaling platforms. In these microdomains, CD44 co-localizes with other receptors, including EGFR, which triggers non-canonical signaling pathways independent of Smads. It has been demonstrated that inhibition of HA synthesis (e.g., by knocking down HAS2 synthase) or blocking CD44 function prevents the activation of α-SMA, even in the presence of TGF-β1, confirming the necessity of the HA-CD44 axis in this process. Moreover, HA level affects the presence of CD44 in lipid rafts, thereby conditioning the efficiency of the entire signal transduction process [175,182,183,184].

Despite lacking its kinase activity, CD44 plays a crucial role in initiating signaling cascades by forming dynamic complexes with other membrane receptors and adaptor proteins. One of the most critical mechanisms of CD44 signaling activation is its functional cooperation with the EGFR, which is conditioned by the presence of HA and TGF-β1. In response to these signals, CD44 and EGFR co-localize in lipid rafts—specialized microdomains of the cell membrane rich in cholesterol and sphingolipids, which are platforms for concentrating signaling components. The formation of the CD44–EGFR complex within lipid rafts is necessary for the activation of intracellular pathways leading to the differentiation of fibroblasts into myofibroblasts [185,186]. Disruption of this organization, by destabilizing rafts or disrupting CD44 binding to HA, results in disorganized signaling and inhibition of further transmission. Signal initiation in rafts first leads to the activation of MAPK/ERK kinase, and then to the activation of calmodulin-dependent kinase II (CaMKII). It has been experimentally demonstrated that ERK phosphorylation is a prerequisite for CaMKII activation, and the blockade of either of these pathways effectively inhibits the expression of myofibroblast markers, such as α-SMA, and prevents complete differentiation. The discussed CD44–EGFR–ERK/CaMKII cascade acts in parallel and synergistically with the classical Smad2/3 pathway, activated by TGF-β receptors. Integrated signaling from both pathways enables the maximal activation of pro-fibrotic genes and the full phenotypic transformation of fibroblasts into contractile, ECM-modifying myofibroblasts [167,176,187,188,189].

Notably, the functionality of lipid rafts appears to be critical for effective CD44-dependent signaling. Restriction of HA synthesis—e.g., by knockdown of the *HAS2* gene—leads to impaired localization of CD44 in these microdomains and reduced ERK/CaMKII signaling. CD44 can also interact with other signaling proteins, such as ERM family proteins (ezrin, radexin, moesin), which link it to the actin cytoskeleton and enhance cellular stability [30].

### 3.2. In Vitro and In Vivo Experimental Evidence for the Role of CD44 in Fibrosis (Lung, Skin, Heart, Liver)

In numerous experimental models, activation of the HA–CD44 pathway has been shown to promote the transformation of fibroblasts into myofibroblasts, as well as to maintain their pro-fibrotic phenotype. This relationship has been confirmed both in vitro studies on fibroblasts isolated from human and animal tissues, as well as in vivo studies in animal models of fibrosis of various organs. Notably, interventions targeting CD44, such as its genetic silencing or antibody blockade, effectively limit collagen deposition, reduce fibroblast activation, and slow the progression of fibrosis (Table 8).

Accumulating preclinical data from various in vitro and in vivo models indicate that modulation of CD44 activity, both through genetic interventions and pharmacological approaches, significantly affects the course and intensity of fibrotic processes in numerous organs. These results support the view that CD44 is not only a marker of the profibrotic phenotype but also plays a role as an actively involved mediator of fibrosis pathogenesis, integrating extracellular with intracellular transduction pathways that lead to fibroblast activation, myofibroblast induction, and the overproduction of ECM components.

In several animal models, the genetic deletion of CD44 (Cd44^−^/^−^) has been shown to attenuate the fibrotic response significantly. For example, Cd44 knockout mice are protected against bleomycin-induced lung fibrosis as well as against myocardial fibrotic remodeling induced by chronic angiotensin II exposure. In both cases, there was a decrease in myofibroblast and macrophage activity, limited collagen accumulation, and reduced expression of pro-fibrotic genes, clearly indicating the crucial role of CD44 in amplifying pro-fibrotic signals in the injured tissue environment [198,199,200].

It is worth emphasizing that similar effects were also obtained using the CD44 immunoneutralization strategy. Administration of monoclonal antibodies directed against CD44 (e.g., clone IM7) to mice resulted in a significant attenuation of the progression of liver and lung fibrosis, which was accompanied by a reduction in the expression of activated myofibroblast markers (e.g., α-SMA) and a decrease in the collagen content in the ECM [201]. Equally promising results were achieved by pharmacological intervention at the level of the CD44 ligand–hyaluronic acid. In models of renal, hepatic, and pulmonary fibrosis, the use of 4-methylumbelliferone (4-MU)—an inhibitor of HA synthesis—led to a reduction in its deposition in the tissue and indirect quenching of CD44 signaling. In particular, in a model of lupus nephritis, both 4-MU and anti-CD44 antibody were shown to reduce the inflammatory infiltrate, limit interstitial fibrosis, and preserve the architecture of the glomeruli and tubules, without affecting the degree of cytotoxic hepatocyte damage or the intensity of inflammation [202,203,204,205]. In addition, in models of fibrosis induced by toxic agents (e.g., CCl_4_) and by hemodynamic factors (e.g., venous congestion in the IVC ligation model), it has been shown that blockade of the HA–CD44 axis translates into limited activation of stellate cells, reduced expression of markers such as S100A4 or TGF-β1, and consequently—limited progression of fibrosis [206]. Therefore, the possibility of therapies targeting the HA–CD44 pathway is on the horizon, whether through the administration of anti-CD44 antibodies, competitive peptides blocking HA binding, inhibition of HA synthesis enzymes, or modulation of alternative CD44 splicing (e.g., variant forms associated with pathology). In diseases such as idiopathic pulmonary fibrosis, where there are currently no effective drugs to reverse the scarring process, intervening in the CD44–HA interaction is a promising new strategy. CD44 has already been proposed as a biomarker of fibrosis activity (e.g., in congestive hepatopathy or scleroderma) and as a target for drugs that inhibit disease progression. High CD44 expression and HA production are observed not only in disease but also in regenerative processes, especially in the skin [207,208]. Therefore, anti-CD44 therapies must be carefully designed to precisely target pathological fibrogenesis niches, thereby limiting long-term CD44 blockade in healthy tissues. Further understanding of the complexity of CD44 signaling (dependent on tissue context and cell type) is needed to develop safe and effective therapeutic approaches. However, the data to date are encouraging—CD44 is emerging as a key regulatory node in the fibrosis signaling network and as a promising target, the modulation of which may benefit patients with progressive fibrotic diseases, for whom treatment options are currently limited.

### 3.3. CD44 and the Invasive Phenotype of Myofibroblasts—Analogies to EMT in Cancer

Advanced stages of fibrotic processes are characterized by the emergence of a population of myofibroblasts with an invasive phenotype, which acquire the ability to migrate actively, degrade the ECM, and penetrate surrounding tissue structures. This aggressive phenotype not only maintains the chronic profibrotic state but also promotes spatial expansion of fibrosis and its progression towards adjacent, previously unchanged organ areas [192,209,210,211].

What is particularly important from the point of view of pathobiological mechanisms is that the behavior of invasive myofibroblasts during advanced fibrosis exhibits striking similarities to the phenotype of cancer cells undergoing EMT. This fundamental phenomenon facilitates the migration, invasion, and formation of metastases by cancer cells. In both cases, increased cell motility, cytoskeletal reorganization, degradation of the basement membrane, and reprogramming of cell adhesion genes, such as E-cadherin, N-cadherin, and vimentin, are observed [212,213,214].

Fibroblasts isolated from the fibrotic lungs of patients with IPF exhibit a highly invasive phenotype that resembles that of EMT cells in terms of their behavior. They can degrade ECM components, cross the basement membrane barrier, and move toward chemotactic gradients—characteristics of transformed cancer cells. A key molecular factor enabling this phenotype is signaling initiated by the HA–CD44 complex. Blocking CD44 or interrupting its interaction with HA (e.g., by using neutralizing antibodies) has been shown to effectively abolish the ability of myofibroblasts to degrade matrix and migrate, thereby inhibiting the progression of fibrosis [214,215,216,217].

An analogous mechanism is observed in the context of malignant tumor progression. During EMT, induced, among other things, by the action of TGF-β1, epithelial cells acquire the features of mesenchymal cells, which allows them to detach from the primary tumor focus and initiate invasion. In many tumor models (including breast, lung, and pancreatic cancer), TGF-β1 has been shown to induce de novo HA synthesis and CD44 expression, which leads to the formation of the HA–CD44 complex and its interaction with kinase receptors such as EGFR. This signaling axis leads to the activation of the PI3K/AKT and MAPK/ERK pathways, as well as cytoskeletal reorganization, resulting in the loss of epithelial features, increased invasiveness, and the acquisition of the complete EMT phenotype [216]. In cancer cells undergoing EMT, experimental inhibition of HA synthesis (e.g., 4-MU) or CD44 silencing (e.g., by RNAi) leads to the suppression of EGFR activity and downstream signaling pathways (ERK, AKT), resulting in the reversal of the EMT phenotype and attenuation of cell invasiveness [218,219,220].

## 4. Impact of Environmental Pollutants on CD44 in Connective Tissue

Environmental factors, including heavy metals, particulate matter, endocrine-disrupting compounds (EDCs), and microplastics, can impact the function and expression of CD44, thereby modulating inflammatory and fibrotic processes in connective tissue. The characteristic tissue response to these factors is chronic damage with concomitant inflammation and extracellular matrix remodeling.

### 4.1. Heavy Metals

Exposure to toxic metals (e.g., lead, cadmium) disrupts the homeostasis of the immune system and connective tissue functions, including the regulation of leukocyte adhesion molecules. It has been observed that chronic exposure to lead leads to a decrease in CD44 expression on the surface of blood cells, for example, in children living in areas contaminated with electronic waste (e-waste). High blood Pb levels have been correlated with lower expression of CD44 and CD58 adhesion molecules on erythrocytes [200,201]. This may impair the ability of blood cells to interact with the endothelium and matrix, weakening proper repair and immune responses. On the other hand, in response to damage caused by heavy metals (such as oxidative stress and cytotoxicity), the body triggers a chronic inflammatory state, characterized by an influx of immune cells. Macrophages and neutrophils accumulating at metal accumulation sites typically exhibit high expression of CD44, which is essential for their adhesion and migration in tissues rich in hyaluronan. It can therefore be assumed that heavy metals indirectly enhance signaling through CD44, e.g., by inducing the release of HA fragments in damaged tissues (HA then acts as a DAMP activating the immune response). The aforementioned population studies in children from e-waste areas have indeed shown an association between increased levels of metals (Pb, Cd) and reduced expression of adhesion molecules (CD44, CD58) on blood cells, which indicates a chronic effect of metals on the hematopoietic and immune systems. In summary, heavy metals can both directly change the level of CD44 on cells (e.g., reduction in CD44 on erythrocytes in lead poisoning) and indirectly, through induced inflammation, increase the demand for CD44 functions in repair processes. Paradoxically, chronic activation of the CD44 pathway can promote uncontrolled fibrosis (e.g., scarring in organs after metal poisoning) [202,203,204].

### 4.2. Particulate Matter (PM)

Air pollutants, especially fine PM_2_._5_ dust particles and crystalline silica (e.g., mine dust), have a documented ability to cause chronic inflammation in the airways and lung fibrosis. CD44 plays a crucial role in the lung’s response to inhaled particles. For example, during exposure to ozone, a strong oxidant present in smog, recruitment of inflammatory cells to the lungs requires the interaction of CD44 with hyaluronic acid. Studies in mice have shown that individuals lacking CD44 are protected from ozone-induced bronchial hyperresponsiveness despite a similar increase in HA concentration in the lungs [205,206,207,208]. This means that damage to lung tissue by ozone leads to the release of HA fragments, which then signal via CD44 on macrophages and neutrophils, facilitating their influx into the alveoli. In the case of silica dust (causing silicosis), chronic inhalation leads to a gradual, fibrotic remodeling of the lungs. As described earlier, this process is mainly dependent on the HA–CD44 pathway. Studies in a mouse model have shown that blockade of CD44 (with the IM7 antibody) alleviates the effects of silica dust, fibroblast activation, and collagen deposition in the lung, and respiratory function is improved. This suggests that dust interaction with the lung initiates an inflammatory-repair cascade mediated by CD44. First, acute inflammation (characterized by the influx of CD44+ cells) occurs, followed by chronic fibrosis (resulting from CD44-dependent activation of fibroblasts). Interestingly, finer dust particles can penetrate deeper into the respiratory system and induce a more severe inflammatory-fibrotic response. It has been found that the smaller the particles (even on the nanoscale), the greater their toxicity and ability to induce fibrosis—this is due to their larger surface area relative to mass and the potential to generate oxidants. The effect, among others, is a stronger polarization of the immune response towards Th2 and more intensive matrix deposition in the lungs after exposure to fine dust. In addition, suspended dust can induce epigenetic changes in cells. For example, PM_2_._5_ particles can modulate DNA methylation and other epigenetic modifications, thereby affecting the expression of various genes. It is possible that this also includes CD44 variant genes, although this aspect requires further research. In summary, environmental particulate matter acts as a trigger for chronic inflammation and fibrosis (especially in the lungs), and CD44 is one of the key receptors involved in the development and maintenance of these pathological processes [192,208,209,210,211].

### 4.3. Endocrine-Disrupting Compounds (EDCs)

EDCs include pesticides, bisphenols (e.g., BPA), phthalates—substances capable of interfering with the endocrine system and causing abnormal metabolic and inflammatory responses. Increasing evidence suggests that exposure to EDCs can stimulate processes characteristic of EMT (epithelial–mesenchymal transition) and fibrosis in various tissues. For example, bisphenol A (BPA) and styrene derivatives can induce EMT in epithelial cells of the respiratory and reproductive systems. Under the influence of these compounds, epithelial cells undergo a transition to a mesenchymal phenotype, becoming more migratory and invasive [211,212]. Since the EMT is accompanied by increased CD44 expression, it is logical to assume that EDCs may secondarily increase the activity of the CD44 pathway in the tissues affected by their action. In addition to inducing EMT, EDCs promote chronic low-grade inflammation [213,214,215]. For example, in people with high blood levels of phthalates, elevated inflammatory markers and matrix remodeling in the liver (nonalcoholic fatty liver disease with fibrosis) are observed [213]. Experimental studies confirm this correlation—chronic exposure to low doses of the phthalate DEHP in mice leads to increased liver damage, inflammatory infiltration (leukocyte infiltration), and increased collagen deposition [214]. Similar changes occur in the adipose tissue of obese people, where some EDCs accumulate. There, increased synthesis of hyaluronic acid and osteopontin was observed, along with enhanced expression of CD44 on macrophages infiltrating adipose tissue [215,216]. Moreover, some EDCs can directly modulate CD44 expression. For example, BPA has been shown to activate the estrogen-related receptor (ERRγ) in A549 lung cancer cells, which is associated with the induction of EMT [218,219]. This suggests that substances with estrogenic activity (such as parabens, alkylphenols) may potentially increase CD44 levels in target cells responding to estrogen signals. Although the effect of specific EDCs on the CD44 pathway requires further study, it is already clear that EDCs contribute to chronic inflammation and fibrosis in tissues, where CD44 acts as a mediator, participating in immune infiltration and phenotypic changes (e.g., EMT) of cells exposed to these compounds.

### 4.4. Microplastics

Microscopic plastic particles (originating from degradation of packaging, textiles, or tire abrasion, among others) are a ubiquitous environmental pollutant. They can enter the body through ingestion or inhalation. It has been proven that the accumulation of microplastics in tissues causes inflammatory reactions and fibrosis and also disrupts the composition of the microbiome [219,220,221]. The immune system recognizes these particles as foreign bodies; at the site of their deposition, macrophage recruitment occurs, and granulomas form (analogous to the inhalation of silica dust or asbestos fibers) [222,223,224,225]. Fibroblasts and myofibroblasts participate in the formation of granulation tissue and the encystation of microplastics, the migration and activation of which depend, among other things, on the interaction of CD44 with the matrix rich in hyaluronan. It can be predicted that in places of microplastic accumulation, there is increased expression of CD44 on macrophages (which form HA-rich granulation tissue) and on fibroblasts that build a fibrous capsule around foreign particles. Long-term presence of microplastics may maintain a chronic inflammation—continuous influx of monocytes and lymphocytes sustains the inflammatory response, which is mediated by signaling through CD44 and its ligands (HA, osteopontin, etc.). Moreover, microplastics often act as carriers for other contaminants (e.g., heavy metals, polychlorinated biphenyls), enhancing the combined toxic effect [226,227,228]. An analogy can be drawn to silicosis—just as silica dust induces lung fibrosis (silicosis) via CD44-dependent mechanisms, microplastics can induce “plasticosis,” characterized by chronic inflammation and scarring of organs (e.g., lungs, gastrointestinal tract) following the accumulation of plastics [229]. This is a newly defined disease term, but case reports (e.g., in seabirds eating plastic) confirm the occurrence of extensive scarring and fibrotic tissue remodeling in the presence of microplastics. This suggests the same molecular pathways as in other forms of chronic inflammation around a foreign body, with a significant contribution from CD44 signaling (Table 9).

## 5. Limitations and Future Research Directions

Despite the abundant evidence indicating the vital role of CD44 in the pathogenesis of tissue fibrosis and inflammatory responses, the current state of knowledge is primarily based on preclinical models, mainly animal and in vitro studies. There is a lack of extensive translational and clinical studies confirming the efficacy and safety of CD44 targeting in humans. Most interventions (e.g., neutralizing antibodies, gene knockouts, or HA synthesis inhibitors) have been performed under controlled conditions, which limits their direct translation into the complex environment of the human body.

In addition, the heterogeneity of CD44 isoforms (CD44s, CD44v3, v6, v7, etc.) and their dynamic expression, which varies depending on the cell type, inflammation, or microenvironment, makes it challenging to determine which form of the molecule is the key pathogenic factor. Most studies do not distinguish between isoforms in analyses, which may mask subtle but critical functional differences.

Another limitation is the insufficient understanding of the long-term effects of modulating the CD44–HA axis. Although inhibition of this pathway is beneficial in fibrosis models, it is unclear whether it also disrupts physiological healing, regeneration, or immune responses. Furthermore, the influence of environmental pollutants on CD44 expression and function has been described mainly in a correlative context. There is a need for cause-and-effect studies that will unequivocally confirm the participation of this receptor in mediating the harmful effects of factors such as microplastics, EDCs, or heavy metals. In light of the growing interest in targeted therapies in chronic diseases, CD44 appears as a promising molecular target in the context of both antifibrotic and immunomodulatory interventions. Future studies should focus on the development of selective inhibitors of CD44 isoforms that will allow for precise modulation of its activity without interfering with the physiological functions of this receptor in regenerative and immune processes. It will also be crucial to develop and validate biomarkers based on CD44 expression profiles—both its membrane and soluble forms (sCD44)—and hyaluronic acid (HA) levels, which will enable dynamic monitoring of fibrosis progression and response to treatment. A key direction of development is the use of combined therapies, including simultaneous inhibition of the CD44–HA axis and canonical TGF-β/Smad pathways, which may yield a synergistic effect in limiting uncontrolled matrix remodeling. It will also be critical to deepen our understanding of the interactions between environmental factors and the regulation of CD44 expression, taking into account the influence of epigenetic modifications and tissue microbiota disorders. Further studies should also include population and clinical assessments of the efficacy and safety of CD44 inhibitors and HA antagonists (such as 4-methylumbelliferone) in patients with idiopathic pulmonary fibrosis, liver cirrhosis or systemic sclerosis. In parallel, it is worth exploring analogies with oncogenesis, including the mechanisms of EMT and acquisition of an invasive phenotype, which may enable translation of therapeutic strategies used in oncology (e.g., anti-CD44 antibodies) to the treatment of fibrogenic diseases. An integrated approach, combining molecular, toxicological, environmental, and clinical studies, may contribute to a more comprehensive understanding of the role of CD44 in connective tissue homeostasis and pathology and initiate a new generation of therapies targeting this multifunctional receptor.

## 6. Conclusions

CD44 is an essential pathophysiological factor in the processes of fibrosis and remodeling of connective tissues. Its activity, dependent on interaction with hyaluronic acid and the presence of specific signaling pathways (EGFR, MAPK, Smad), affects fibroblast differentiation, inflammatory cell migration, myofibroblast formation, and intensification of the inflammatory response. Experimental models of lung, skin, heart, and liver fibrosis demonstrate that genetic deletion of CD44, its pharmacological blockade, or inhibition of HA biosynthesis leads to inhibition of the progression of fibrogenic changes. At the same time, the similarity between the phenotypes of invasive myofibroblasts and cancer cells after EMT emphasizes the commonality of mechanisms driving aggressive tissue remodeling. Finally, environmental factors—from heavy metals to microplastics—modulate CD44 activity and may enhance its pathogenic effect by stimulating inflammation, EMT, and matrix overproduction. The collected data identify CD44 as a potential target for therapeutic interventions in fibrotic diseases, as well as a biomarker for assessing exposure to environmental factors.

## Figures and Tables

**Table 1:** Characteristics of selected CD44 variants and their potential biological significance (based on [30,31]). This table summarizes key variants of the CD44 gene, detailing their specific amino acid changes, structural locations, and functional relevance to various biological processes. CD44, a transmembrane glycoprotein, plays a crucial role in cell adhesion, migration, and the regulation of immune responses. The variants presented here highlight the diversity in CD44 structure and its potential implications in immunohematology, cell signaling, and receptor-ligand interactions. Each variant is associated with specific regions of the protein, ranging from the extracellular domain to the transmembrane region, and may influence its function in different physiological and pathological contexts.

Variant (dbSNP ID)	Amino Acid Change	Structural Location	Functional Description and Biological Relevance
VAR_006490 (rs3694738421)	Arg46Pro (arginine → proline)	Extracellular domain (N-terminal)	This variant underlies the Indian (In) blood group system and differentiates In^a^ and In^b^ antigens on erythrocytes. The proline-coding allele determines the rare In^a^ antigen, which may elicit alloimmune transfusion reactions. It is not pathogenic in systemic health but has significant clinical relevance in immunohematology.
VAR_030325 (rs11607491)	Substitution at position 393	Extracellular domain	Although its functional consequences are not fully characterized, the variant is located in the ligand-binding region and may affect CD44’s interaction with hyaluronan or extracellular matrix components.
VAR_021147 (rs96666074)	Substitution at position 417	Extracellular domain	Located near known glycosylation sites, this substitution may alter CD44 glycosylation patterns, potentially impacting receptor-ligand interactions or immune recognition.
VAR_030326 (rs14675589)	Substitution at position 479	Juxtamembrane region (extracellular–transmembrane junction)	This variant may affect the membrane topology of CD44 and its susceptibility to proteolytic cleavage (shedding), thereby influencing the levels of soluble CD44 (sCD44) and downstream signaling.
VAR_030327 (rs122733971)	Substitution at position 494	Transmembrane domain or adjacent region	This mutation may modulate the anchoring of CD44 in the membrane and alter its interactions with neighboring receptors or lipid rafts, influencing receptor clustering and signaling efficiency.

**Table 2:** Detailed table showing known CD44 protein ligands, grouped by molecule type and their primary biological functions (based on [4,34,35,36,37,38,39,41,42]). The table highlights the diversity of CD44 ligands, ranging from glycosaminoglycans and extracellular matrix proteins to adhesive proteins, growth factors, proteases, and acute phase proteins. These interactions emphasize the pleiotropic functions of CD44 in cell adhesion, migration, proliferation, differentiation, immune response modulation, and tumor progression. Furthermore, the compilation demonstrates how CD44 can act as an integrative receptor that organizes ligand complexes and facilitates downstream signaling pathways such as PI3K, MAPK, and Rho-GTPases.

Ligand Type	Ligand Name	Description and Biological Functions
Glycosaminoglycans (GAGs)	Hyaluronic acid (HA)	Primary ligand of CD44; regulates adhesion, migration, proliferation, differentiation, and inflammatory response
	Heparan sulfate (HS)	Facilitates the binding of growth factors, present in proteoglycan forms of CD44
	Chondroitin sulfate (CS)	Associated with the CD44v isoform; influences interactions with the ECM
Extracellular matrix proteins	Osteopontin (OPN, SPP1)	Modulation of cell migration and adhesion; expression in inflammation and tumorigenesis
	Fibronectin (FN)	Adhesion and signaling; interacts with integrins and CD44
	Laminin	Supports epithelial cell interactions with the basement membrane
	Collagen type I, II, III, IV	Effect on cell adhesion, migration, and invasion
Adhesive proteins	Selectins (L-, E-selectin)—via HCELL form	Participation in leukocyte rolling is meaningful in the immune response
	ICAM-1 (ang. intercellular adhesion molecule 1)	Supporting role in lymphocyte transmigration
Signaling proteins and growth factors	TGF-β, HGF, VEGF, EGF—indirectly through complexes with HA or heparan	CD44 enables local presentation and concentration of growth signals
Proteases	Matrix metalloproteinases (MMP-2, MMP-9)	Interactions with CD44 promote ECM degradation and cell migration.
Acute phase proteins	Pentraxin 3 (PTX3)	Regulation of inflammatory response, interaction with HA and CD44
Other	Serpins, complement proteins	Less frequently described ligands: immunological and proteolytic significance
	Receptors and co-receptors (e.g., EGFR, TGF-βR)—signaling complexes	They cooperate with CD44 in the activation of PI3K, MAPK, and Rho-GTPase pathways

**Table 3:** Characteristics of ERM family proteins in the context of interaction with CD44 (based on [51,86,87,88,89,90]). The table summarizes structural and functional features of ezrin, radixin, and moesin, emphasizing their subcellular localization and roles in linking the actin cytoskeleton with the plasma membrane. Each ERM protein directly interacts with CD44, stabilizing its position within microvilli, intercellular junctions, or migratory structures, thereby facilitating adhesion, signaling, and cytoskeletal reorganization. The functional consequences of these interactions include the regulation of cell polarity, migration, inflammatory responses, and the mechanical coupling of adhesion with intracellular signaling. Dysfunctions of ERM–CD44 interactions are associated with diverse pathologies such as cancers, viral infections, chronic inflammation, and hereditary disorders (e.g., hearing loss linked to RDX mutations).

Feature	Ezrin	Radixin	Moesin
Gene symbol (human)	EZR	RDX	MSN
Subcellular localization	Cell membrane, microvilli, surface of cellular projections	Intercellular junctions, cortical cytoplasm	Microvilli, lamellipodia, and ECM contact zones
Biological function	Links the actin cytoskeleton to the plasma membrane; involved in shaping microvilli and cell adhesion	Stabilizes the plasma membrane and intercellular junctions; regulates membrane elasticity	Regulates cell shape, leukocyte transmigration, and inflammatory response
Interactions with CD44	Direct interaction with CD44’s cytoplasmic domain; involved in targeting CD44 to microvilli and lipid rafts	Anchors CD44 at sites of intercellular contact and cytoskeletal reorganization	Stabilizes CD44 in migrating immune cells; involved in Rho/Rac pathway activation
Functional significance of CD44–ERM	Integrates extracellular signals with intracellular responses; mechanical coupling of adhesion and signaling	Maintains cell polarity and membrane tension; supports CD44 signaling	Enhances pro-inflammatory response; involved in cytoskeletal reorganization during migration
Diseases associated with dysfunction	Cancers (e.g., gastric cancer, leukemias), kidney diseases, viral infections (e.g., HIV, EBV)	Hearing loss (RDX mutations), liver and gallbladder cancers	Chronic inflammatory conditions, lymphomas, head and neck cancers

**Table 4:** Summary of CD44 Domain Structure (Canonical Isoform) [8]. The table outlines the structural organization of CD44, dividing it into extracellular, transmembrane, and cytoplasmic domains. The extracellular domain mediates binding to hyaluronic acid and other ECM ligands and undergoes glycosylation, while the transmembrane segment anchors the receptor in the plasma membrane and defines its orientation. The cytoplasmic domain interacts with ERM proteins, undergoes regulatory phosphorylation, and transduces signals that connect extracellular cues with intracellular responses. Together, these domains provide the structural and functional basis for CD44’s role as an adhesion molecule and signaling platform.

Segment	Amino Acid Range	Function
Extracellular domain	21–649	Binding to hyaluronic acid (HA) and other ECM ligands; glycosylation modifications.
Transmembrane segment	650–670	Anchoring in the membrane, defining N-/C-terminal orientation
Cytoplasmic domain	671–742	Interactions with ERM proteins, signal transduction, and regulatory phosphorylation

**Table 6:** Overview of key PTMs of CD44 (based on [128,130,131,132,133,134,135,136,137,138,139,140]). The table summarizes major post-translational modifications (PTMs) of CD44, including glycosylation, phosphorylation, proteolytic cleavage, and ubiquitination. These modifications occur in distinct domains of the receptor and regulate folding, stability, ligand binding, receptor activation, and intracellular trafficking. Collectively, PTMs fine-tune CD44 functions by modulating adhesion, migration, cytoskeletal reorganization, and signal transduction. Dysregulation of these processes contributes to altered immune responses, tumor progression, and metastasis, underlining the biological relevance of PTMs in CD44-mediated pathways.

Type of Modification	Location/Target Residues	Functional Description	Biological Significance
N-glycosylation	Asn within the Asn-X-Ser/Thr motif (e.g., in the LINK domain)	Attachment of N-glycans	Essential for proper folding, stability, and affinity for HA
O-glycosylation	Ser/Thr (especially in variable splice regions, e.g., Thr-637/638)	Addition of short sugar chains and glycosaminoglycans (e.g., chondroitin sulfate)	Modulates ligand recognition, protects from proteolysis, and affects cell migration
Phosphorylation	Ser-672, Ser-706; other Ser/Thr residues in the C-terminal tail	Regulates receptor activation status and interactions with adaptor proteins (e.g., ERM)	Alters adhesion and migration signaling, influences cytoskeletal organization
Proteolysis (shedding)	Near the transmembrane domain (extracellular side)	Cleavage of the extracellular domain by MMPs and other proteases	Reduces surface CD44 expression, generates soluble form (sCD44), and is potentially further cleaved by γ-secretase and nuclear signaling
Ubiquitination	Lys-704, Lys-715 (in the cytoplasmic tail)	Covalent modification affecting intracellular trafficking and degradation	May regulate receptor abundance and endosomal sorting

**Table 8:** Experimental evidence of CD44 involvement in organ fibrosis (based on [6,20,128,191,192,193,194,195,196,197]). The table compiles preclinical and translational studies that demonstrate the role of CD44 in fibrotic remodeling across multiple organs. In pulmonary fibrosis, the HA–CD44 axis drives fibroblast invasiveness, while genetic or antibody-mediated blockade of CD44 markedly reduces fibrotic progression. In the skin, altered CD44 expression and elevated soluble CD44 levels correlate with disease severity, suggesting both pathogenic and protective roles depending on the context. Cardiac and hepatic fibrosis models further highlight CD44 as a mediator of myofibroblast activation, collagen deposition, and tissue remodeling, positioning it as a key integrator of inflammatory and fibrotic signals and a potential therapeutic target. ↑ increased level/increased expression.

Organ	Experimental Model	Key Observations	Functional Conclusions
Lungs (IPF)	Bleomycin-induced pulmonary fibrosis in mice; HAS2 overexpression; CD44 knockout	HAS2↑ → severe fibrosis; HAS2 KO protects against fibrosis CD44 KO–loss of invasive fibroblast phenotype and fibrosis suppression Anti-CD44 antibody alleviates pulmonary changes IPF fibroblasts: high CD44-dependent invasiveness	The HA–CD44 axis is essential for the pro-fibrotic phenotype in the lungs; CD44 blockade reduces fibrosis and fibroblast invasiveness.
Skin (SSc)	Patient-derived fibroblasts from systemic sclerosis; sCD44 concentration analysis; wound healing in CD44 KO mice	Altered CD44 expression in fibroblasts Increased sCD44 correlates with a milder clinical phenotype (less skin/lung involvement) CD44 KO–reduced collagen and slower scar formation	CD44 may support both wound healing and pathological skin sclerosis; sCD44 potentially exerts protective effects
Heart	Ang II-induced fibrosis; pressure overload model; CD44 KO	Ang II–increased CD44 expression + TNFα/NF-κB activation + macrophage migration CD44 KO–less collagen, reduced myofibroblast proliferation CD44 blockade–attenuated post-infarction remodeling, improved cardiac function	CD44 mediates cardiac remodeling by integrating inflammatory and fibrotic signals; its inhibition halts fibrosis progression
Liver	Hepatic congestion model (IVC ligation); CD44 and HA immunohistochemistry; CD44 neutralization (IM7)	CD44↑ and HA↑ in hepatic stellate cells CD44 blockade–reduced collagen (Sirius red, hydroxyproline) and S100A4 expression ALT and inflammatory cytokines unchanged–effect independent of hepatocyte injury	CD44 activates stellate cells and promotes fibrosis independently of inflammation; it represents a promising therapeutic target and biomarker

**Table 9:** Impact of environmental pollutants on the CD44–HA pathway in connective tissue (based on [210,211,212,213,214,215,216,217,218,219,220,221,222,223,224,225,226,227,228,229]). The table summarizes experimental and clinical evidence on how diverse environmental pollutants influence the CD44–hyaluronic acid (HA) axis in connective tissue remodeling. Heavy metals impair cell adhesion molecule expression on erythrocytes but enhance CD44 expression on immune cells, thereby promoting chronic inflammation and fibrosis. Particulate matter (PM_2_._5_, silica dust) relies on CD44 for fibroblast activation and inflammatory cell recruitment, driving lung fibrosis, while endocrine-disrupting chemicals (EDCs) such as bisphenol A and phthalates induce CD44-dependent EMT, collagen deposition, and inflammatory infiltration. Microplastics further activate CD44 on macrophages and fibroblasts, supporting granuloma formation and fibrosis, and may act as carriers of toxic substances, amplifying tissue damage. Collectively, these findings underscore CD44–HA as a central mediator linking environmental exposure to chronic inflammation, fibrotic remodeling, and impaired tissue repair.

Type of Pollutant	Impact on CD44 and HA	Effects in Connective Tissue	Molecular Mechanisms/Experimental Data
Heavy metals *(lead, cadmium)*	• Decreased expression of CD44 and CD58 on erythrocytes (e.g., in children exposed to e-waste) • Increased CD44 expression on macrophages and neutrophils at metal accumulation sites	• Impaired reparative and immune functions • Chronic inflammation • Enhanced organ fibrosis	• Lead reduces cell adhesion molecule expression (CD44/CD58), impairing ECM-cell interactions • HA fragmentation acts as a DAMP, activating immune responses via CD44
Particulate matter *(PM_2.5_, silica dust)*	• CD44 mediates the recruitment of inflammatory cells to the lungs in response to ozone • CD44 blockade in silicosis models reduces collagen deposition	• Chronic lung inflammation • Pulmonary fibrosis (e.g., silicosis, fibrosing alveolitis) • Bronchial hyperreactivity	• CD44 loss protects against ozone-induced response (despite HA increase) • CD44 is essential for fibroblast activation and inflammatory cell migration • PM nanoparticles → oxidative stress, Th2 polarization, epigenetic activation of pro-fibrotic genes
Endocrine-disrupting chemicals (EDCs) *(bisphenol A, phthalates, pesticides)*	• Induction of CD44 expression in epithelial cells undergoing EMT • Increased CD44 on tissue macrophages (e.g., in adipose tissue)	• EMT and mesenchymal transition • Chronic inflammation • Fibrosis (e.g., in liver, lung, adipose tissue)	• BPA activates ERRγ → EMT in A549 cells • Phthalates increase collagen deposition and leukocyte infiltration in the liver • EDCs enhance HA and osteopontin production—both CD44 ligands
Microplastics	• Induce CD44 expression on macrophages and fibroblasts surrounding particles • Indirect CD44 activation via DAMPs and cytokines	• Granuloma formation around microplastics • Chronic inflammation and fibrosis in lungs, liver, intestines (“plasticosis”)	• CD44- and HA-dependent migration and activation of myofibroblasts • Microplastic particles act as toxin carriers (e.g., metals, PCBs), amplifying inflammatory response • Mechanism

## Data Availability

No new data were generated or analyzed in this study. All data cited and discussed are publicly available from the original publications referenced in the manuscript.

## Abbreviations

The following abbreviations are used in this manuscript:

CD44	Cluster of Differentiation 44
HA	Hyaluronic Acid
TGF-β	Transforming Growth Factor Beta
EGF	Epidermal Growth Factor
VEGF	Vascular Endothelial Growth Factor
PI3K	Phosphoinositide 3-Kinase
AKT	AKT Serine/Threonine Kinase
MAPK	Mitogen-Activated Protein Kinase
RhoA	Ras Homolog Family Member A
ROCK	Rho-associated Protein Kinase
ERM	Ezrin–Radixin–Moesin
EMT	Epithelial–Mesenchymal Transition
CSC	Cancer Stem Cell
ECM	Extracellular Matrix
MMP	Matrix Metalloproteinase
STAT3	Signal Transducer and Activator of Transcription 3
NF-κB	Nuclear Factor Kappa-light-chain-enhancer of Activated B Cells
ALT	Alanine Aminotransferase
SSc	Systemic Sclerosis
IPF	Idiopathic Pulmonary Fibrosis
RZS	Rheumatoid Arthritis (RA)
LN	Lupus Nephritis
EDCs	Endocrine Disrupting Chemicals
BPA	Bisphenol A
DEHP	Di(2-ethylhexyl) Phthalate
ERRγ	Estrogen-Related Receptor Gamma
PM_2_._5_	Particulate Matter ≤ 2.5 µm
4-MU	4-Methylumbelliferone
OS	Overall Survival
KO	Knockout
sCD44	Soluble CD44
DAMP	Damage-Associated Molecular Pattern
PCB	Polychlorinated Biphenyls
